# Skin and soft tissue infection incidence before and during the COVID-19 pandemic

**DOI:** 10.1017/S0950268823001802

**Published:** 2023-11-06

**Authors:** Prudencio Merino, Deborah Kupferwasser, Evelyn A. Flores, Donna Phan Tran, Abisay Ortega, Loren G. Miller

**Affiliations:** 1 Division of Infectious Diseases, The Lundquist Institute at Harbor-UCLA Medical Center, Torrance, CA, USA; 2 David Geffen School of Medicine, Los Angeles, CA, USA

**Keywords:** COVID-19, skin infections, soft tissue infections

## Abstract

Skin and Soft Tissue Infections (SSTIs) are common bacterial infections. We hypothesized that due to the COVID-19 pandemic, SSTI rates would significantly decrease due to directives to avoid unneeded care and attenuated SSTIs risk behaviours. We retrospectively examined all patients with an ICD-10 diagnosis code in the Los Angeles County Department of Health Services, the second largest U.S. safety net healthcare system between 16 March 2017 and 15 March 2022. We then compared pre-pandemic with intra-pandemic SSTI rates using an interrupted time series analysis. We found 72,118 SSTIs, 46,206 during the pre-pandemic period and 25,912 during the intra-pandemic period. Pre-pandemic SSTI rate was significantly higher than the intra-pandemic rate (3.27 vs. 2.31 cases per 1,000 empanelled patient-months, *P* < 0.0001). The monthly SSTI cases decreased by 1.19 SSTIs/1,000 empanelled patient-months between the pre- and intra-pandemic periods (*P* = 0.0003). SSTI subgroups (inpatient, observation unit, emergency department, and outpatient clinics), all had significant SSTI decreases between the two time periods (*P* < 0.05) except for observation unit (*P* = 0.50). Compared to the pre-COVID-19 pandemic period, medically attended SSTI rates in our large U.S. safety net healthcare system significantly decreased by nearly 30%. Whether findings reflect true SSTI decreases or decreased health system utilization for SSTIs requires further examination.

Skin and soft tissue infections (SSTIs) represent a group of infections ubiquitous in clinical care [1]. Among bacterial infections occurring in the ambulatory setting, SSTI incidence in the United States is high, with 4.85 cases per 100 persons per year [1, 2], exceeding the incidence of pneumonia and urinary tract infections [3]. Furthermore, SSTIs in the United States alone account for more than 14 million annual visits to a healthcare facility for treatment [4, 5]. Of note, approximately 95% of SSTIs are managed in the outpatient setting [3].

Globally, the severe acute respiratory syndrome coronavirus 2 (SARS-CoV-2) pandemic drove a great demand for healthcare services [6]. As SARS-CoV-2 spread throughout the United States, the delivery of acute care services shifted to accommodate the surge of patients being hospitalised for COVID-19 [7]. The drastic shift in healthcare delivery patterns meant that patients seeking care for non-COVID-19 conditions were severely impacted [8]. As emergency department (ED) visits and hospitalisations increased for patients with COVID-19 infections, ED visits and hospital admissions for non-COVID-19 conditions significantly decreased, falling 38% in 2020 compared with 2019 in countries such as the United States and Italy [9, 10], suggesting that patients with non-COVID-19 conditions were not seeking care during the pandemic.

There are limited data on the effect of the pandemic on common bacterial infections, specifically SSTIs. An Italian study reported an 80% decrease in ED visits for SSTIs during a pandemic lockdown in early 2020 [6]. However, this decrease may not be consistent with changes in ED visit for SSTIs in other countries, given that public health messaging, pandemic response, and healthcare delivery systems differ widely between countries. To understand the impact that the COVID-19 pandemic has on care sought for a common bacterial infection, specifically SSTIs, we performed a retrospective study in a large U.S. safety net hospital system. We hypothesised that due to the COVID-19 pandemic and associated changes in patient care-seeking behaviour and/or behaviours that drive SSTIs such as person–person contact, SSTI rates would decrease during the pandemic.

We performed a health system-wide retrospective study, using the Los Angeles County Department of Health Services (DHS) patient electronic medical record databases between 16 March 2017 and 15 March 2022. The DHS is the second-largest U.S. safety net hospital system consisting of four major medical centres and 20 clinics or ambulatory care centres.

To identify SSTI cases, we used the International Classification of Diseases, 10th Revision, Clinical Modification (ICD-10) using SSTI codes, as described previously [3] (Table 1). We also extracted information on the location of care (inpatient, observation or short care stay unit, ED, and outpatient clinic). SSTI cases from 16 March 2017 through 15 March 2020 (36-month period) were considered ‘pre-pandemic’. Cases from 1 April 2020 to 31 March 2022 (24-month period) were considered ‘intra-pandemic’. We chose to use entire year time periods given the seasonality of SSTIs. We did not analyse the time period between 16 March and 31 March 2020, given that this time period was likely a transition between the pre-pandemic and intra-pandemic time periods (Figure 1). In particular, given that SSTIs typically take time between skin injury and disease manifestation, we thought this period could represent both SSTIs acquired during the pre-pandemic period and thus excluded it from the analysis.

**Table 1.** Skin and soft tissue infections (SSTIs) by clinical condition and ICD-10 code

Description	ICD-10 codes
Cutaneous abscess, furuncle, and carbuncle	L02.xxx
Cellulitis and acute lymphangitis	L03.xxx
Other local infections of skin and subcutaneous tissue	L08.x
Erysipelas	A46
Mastitis	O91.xx, N61.0, N61.1

**Figure 1.**
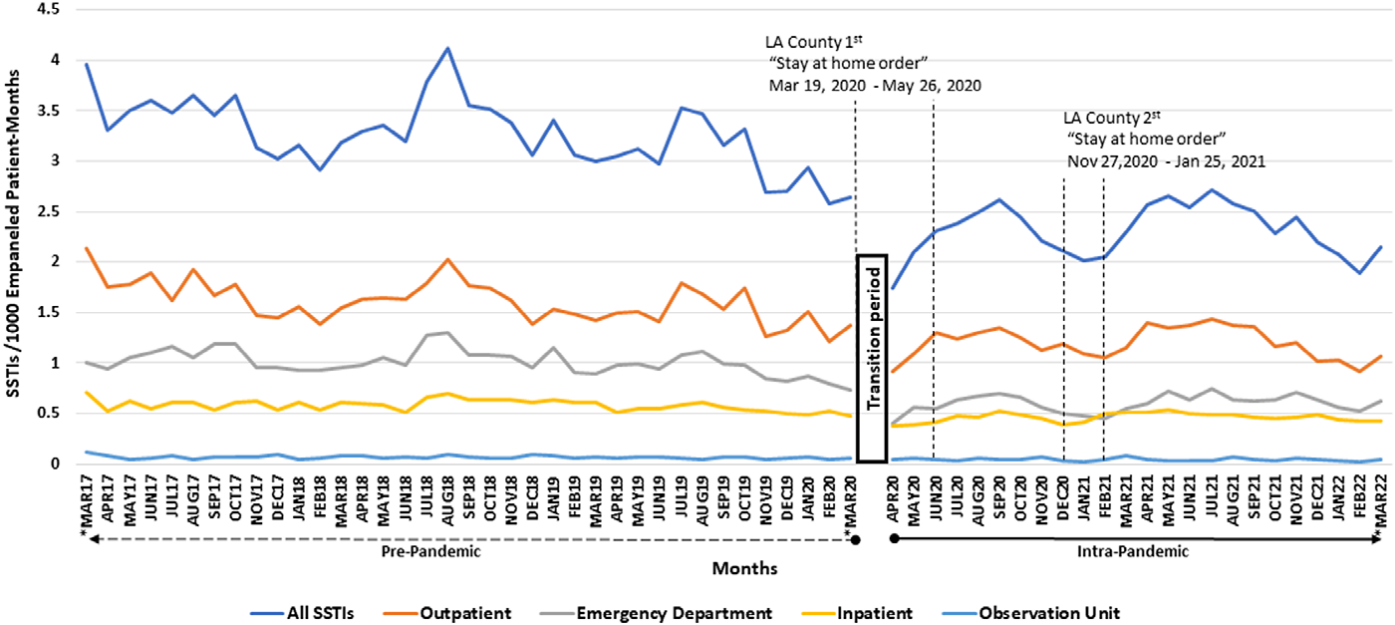
SSTI rates (SSTIs/1000 empaneled patient-months) for pre-pandemic and intra-pandemic periods are graphically represented. Immediately about the X-axis label, the horizontal dashed line pointing left indicates the pre-pandemic period and black solid line pointing right indicates the intra-pandemic period. The first set of vertical perpendicular dashed lines represent the interval period of the first Los Angeles County Department of Health Services (LAC DHS) “stay at home order”. The second set of vertical perpendicular dashed lines represent the interval period of the second (LAC DHS) “stay at home order”. The Gap between MAR20 (March 2020) and APR20 (April 2020) is the transition period between pre-pandemic and intra-pandemic, which we censored (see text). The asterisks below MAR17, MAR20, and MAR22 were used to indicate that we used only half months of data in order to keep the time periods at intervals of 1 calendar year (see text for details).

To define an individual SSTI event, we used prior SSTI definitions that used electronic health records [3]. In particular, any two or more consecutive encounters with SSTIs or SSTI complications within 30 days of each other were deemed part of the same SSTI event. SSTI encounters separated by ≥31 days were considered separate SSTI events. If care locations for an SSTI occurred at >1 site (e.g. ED and outpatient), we categorised that event’s location based on the highest level of care using the following hierarchy (from lowest level of care to highest): outpatient clinics, ED, observation unit, and inpatient. For those with SSTI encounters <30 days apart that were combined into a single SSTI event, the earliest encounter data were considered the onset date.

Overall, SSTI rates were obtained from the overall empanelled population in the DHS system for each month within the study period and used as our denominator. The empanelled population is those people enrolled in the health plans that contract with DHS clinics and hospitals. Our rates were expressed as SSTIs/1,000 empanelled patient months. For fractions of months, we normalised SSTI rates to express monthly rates.

We calculated mean SSTI rates on a monthly basis for each care location and overall DHS-wide rates. Monthly mean pre-pandemic rates vs. mean intra-pandemic SSTI rates were compared using an interrupted time-series analysis.

All data obtained from DHS databases were handled in compliance with the Health Insurance Portability and Accountability Act (HIPPA). The study was approved as an exempt investigation by the Institution Review Board at the Lundquist Institute for Biomedical Innovation at Harbor-UCLA Medical Center. All data analysis was performed using SAS Software v9.4 (SAS Institute, Cary, NC).

During our study period, we found a total of 72,118 SSTIs. Most SSTIs were seen in an outpatient setting (36,167), followed by the ED (21,035), inpatient (13,433), and observation unit (1,483). In the pre-pandemic period and intra-pandemic period, there were 46,206 and 25,912 SSTIs, respectively. Using an interrupted time-series analysis for single series and comparative design, during the COVID-19 pandemic, the number of monthly SSTI cases decreased by 1.19 SSTIs/1,000 empanelled patient months (*P* = 0.0003). The interrupted time-series analysis found that during the pre-pandemic period, the monthly rate of change in SSTIs significantly decreased by 0.019 SSTIs/1,000 empanelled patient months (*P* = 0.0001), and during the intra-pandemic, it significantly increased by 0.020 SSTIs/1,000 empanelled patient months per month period (*P* = 0.02). The interrupted time-series analysis also found that during the COVID-19 pandemic transition period (Figure 1), there was a significant decrease of 1.37 SSTIs/1,000 empanelled patient months (*P* = 0.0008) from the pre-pandemic to intra-pandemic periods.

For outpatient care, in the pre-pandemic period, there were 22,699 SSTIs and there were 13,468 SSTIs during the intra-pandemic period. Using an interrupted time-series analysis for single series and comparative design, during the COVID-19 pandemic, the number of monthly SSTI cases significantly decreased by 0.44 SSTIs/1,000 empanelled patient months (*P* = 0.02). We found that during the pre-pandemic period, the monthly rate of change in SSTIs significantly decreased by 0.011 SSTIs/1,000 empanelled patient months (*P* = 0.0001), but the increase during the intra-pandemic was not significant by 0.009 SSTIs/1,000 empanelled patient months per month period (*P* = 0.08). We also found that during the COVID-19 pandemic transition period, there was a significant decrease of 0.51 SSTIs/1,000 empanelled patient months from the pre-pandemic to intra-pandemic periods (*P* = 0.03).

In the ED, there were 14,317 SSTIs and 6,718 SSTIs during the pre-pandemic and intra-pandemic periods, respectively. During the COVID-19 pandemic, the number of monthly SSTI cases significantly decreased by 0.55 SSTIs/1,000 empanelled patient months (*P* < 0.0001). We found that during the pre-pandemic period, the monthly rate of change in SSTIs significantly decreased by 0.005 SSTIs/1,000 empanelled patient months (*P* = 0.004), and during the intra-pandemic, it significantly increased by 0.007 SSTIs/1,000 empanelled patient months per month period (*P* = 0.01). We also found that during the COVID-19 pandemic transition period (Figure 1), there was a significant decrease of 0.61 SSTIs/1,000 empanelled patient months from the pre-pandemic to intra-pandemic periods (*P* < 0.0001).

For the observation unit, there were 957 SSTIs and 526 SSTIs during the pre- and intra-pandemic periods. During the COVID-19 pandemic, the number of monthly SSTI cases decreased non-significantly by 0.011 SSTIs/1,000 empanelled patient months (*P* = 0.50). We found that during the pre-pandemic period, the monthly rate of change in SSTIs non-significantly decreased by 0.0004 SSTIs/1,000 empanelled patient months (*P* = 0.06) and non-significantly decreased during the intra-pandemic by 0.00007 SSTIs/1,000 empanelled patient months per month period (*P* = 0.88). We also found that during the COVID-19 pandemic transition period (Figure 1), there was a non-significant decrease of 0.011 SSTIs/1,000 empanelled patient months from the pre-pandemic to intra-pandemic periods (*P* = 0.58).

Finally, for inpatients, there were 8,233 SSTIs and 5,200 SSTIs during the pre- and intra-pandemic periods, respectively. During the COVID-19 pandemic, the number of monthly SSTI cases significantly decreased by 0.23 SSTIs/1,000 empanelled patient months (*P* < 0.0001). We found that during the pre-pandemic period, the monthly rate of change in SSTIs significantly decreased by 0.002 SSTIs/1,000 empanelled patient months (*P* = 0.006), and during the intra-pandemic, it significantly decreased by 0.004 SSTIs/1,000 empanelled patient months per month period (*P* = 0.006). We also found that during the COVID-19 pandemic transition period (Figure 1), there was a significant decrease of 0.27 SSTIs/1,000 empanelled patient months from the pre-pandemic to intra-pandemic periods (*P* = 0.0002).

Overall, in our retrospective U.S.-based study of a large safety net population, we saw a decrease in medically attended SSTIs across all sites of care during the COVID-19 pandemic period compared with pre-pandemic times. The reason for this decrease cannot be determined from our data but may be associated with a fear of becoming infected with SARS-CoV-2 by acquiring the virus in a high-risk setting such as a healthcare facility [9]. Our findings may also suggest that persons who had an SSTI may have avoided seeking medical care and managed the infection at home. In addition, it is possible that ‘stay-at-home orders’ set by the Los Angeles County Department of Public Health played a role in the decrease in SSTIs due to the decrease in activities that predispose persons to SSTIs, such as sports and physical laboured jobs.

We observed that SSTI rates within the pandemic period dropped significantly during the COVID-19 pandemic at all levels of healthcare delivery, that is clinics, EDs, and acute care patients. Interestingly, the magnitude of decrease was greatest in the ED (45% decrease), compared with the observation unit (31% decrease), outpatient setting (25% decrease), and inpatient setting (21% decrease). These results suggest that the effect of the pandemic decreased medically attended SSTIs, which disproportionally prevented less severe SSTIs but had a lesser impact on severe SSTIs.

Examining trends within the pandemic period, a more pronounced SSTI rate decrease can be seen during the Los Angeles County Department of Health Services ‘stay-at-home orders’ that took place mostly during the intra-pandemic period on 19 March 2020 through 26 May 2020 and 27 November 2020 through 25 January 2021 (Figure 1). This finding suggests that the stay-at-home orders had major effects on medically attended SSTIs than during times without stay-at-home orders. Interestingly, SSTI rates in our healthcare systems after the second ‘stay-at-home order’ in February 2021 did not return to historical levels (i.e. from March 2017 through March 2020). In particular, the post-February 2021 SSTI rate was 2.38 (range 1.89–2.71) compared with the historical average of 3.27 (range 2.58–4.12), *P* < 0.0001, in a post hoc analysis using a t-test.

Others have examined the impact that the pandemic had on various medical conditions during the COVID-19 pandemic compared with pre-pandemic time periods. For example, Santi et al. found a reduction in care visits in Italy for non-COVID-19 conditions such as skin and subcutaneous tissue conditions, cardiovascular diseases, ischaemic cerebrovascular disorders, and ischaemic heart disease [6]. Ojetti et al. [9] found a 38% decrease in emergency department admissions for non-COVID-19 conditions in Italy between January and March 2020 (when SARS-CoV-2 was circulating locally) compared with 2019. Similarly, Jeffery et al. [7] found that during the first four months of 2020, emergency department visits decreased by more than 40% in five independent healthcare systems in five U.S. states compared with the number of emergency department visits before the COVID-19 pandemic.

Our study has limitations. First, this is a retrospective study using registry data relying on accurate documentation in medical charts. The accuracy of SSTI determination using ICD-10 codes is unclear. However, we would not expect inaccuracies of SSTI diagnosis to preferentially inflate or deflate rates differentially in the pre-pandemic or intra-pandemic time periods. In addition, we measured medically attended SSTIs, which may or may not reflect the true number of SSTIs in our patient population. Due to the pandemic, it is possible that individuals with SSTIs may not have sought care for their SSTIs due to stay-at-home orders, fear of contracting COVID-19 in a medical setting, as noted above, and perhaps managing less severe infections at home. Thus, we might have substantially underestimated true SSTI rates during the pandemic.

The strengths of this study are the large population and time frame we analysed. Studies comparing pre-pandemic and intra-pandemic periods in early 2020 typically examined the first few weeks before and after lockdown periods [6, 7, 9]. In our study, we analysed robust time periods, that is 36 months before and 24 months during the pandemic period. We were also able to show the effects of the pandemic through different sites of care delivery for SSTIs.

In summary, our analysis showed a significant decrease in medically attended SSTI rates during the COVID-19 pandemic period in our large safety net hospital system. These decreases occurred at all levels of care delivery. Understanding the effects of pandemic restrictions on patient care for common acute bacterial infections such as SSTIs can help policymakers anticipate expected trends in healthcare access for acute bacterial infections in healthcare utilisation when future pandemics occur.

## Data Availability

Data from this analysis are available, upon request, from the corresponding author.
